# Identifying Odd/Even-Order Binary Kernel Slices for a Nonlinear System Using Inverse Repeat m-Sequences

**DOI:** 10.1155/2015/454638

**Published:** 2015-03-22

**Authors:** Jin-yan Hu, Gang Yan, Tao Wang

**Affiliations:** School of Biomedical Engineering, Southern Medical University, Guangzhou, Guangdong 510515, China

## Abstract

The study of various living complex systems by system identification method is important, and the identification of the problem is even more challenging when dealing with a dynamic nonlinear system of discrete time. A well-established model based on kernel functions for input of the maximum length sequence (m-sequence) can be used to estimate nonlinear binary kernel slices using cross-correlation method. In this study, we examine the relevant mathematical properties of kernel slices, particularly their shift-and-product property and overlap distortion problem caused by the irregular shifting of the estimated kernel slices in the cross-correlation function between the input m-sequence and the system output. We then derive the properties of the inverse repeat (IR) m-sequence and propose a method of using IR m-sequence as an input to separately estimate odd- and even-order kernel slices to reduce the chance of kernel-slice overlapping. An instance of third-order Wiener nonlinear model is simulated to justify the proposed method.

## 1. Introduction

Living systems usually exhibit complex and nonlinear behaviors [1–3], which can be characterized by a mathematical model carefully tuned to represent the relationship between the input and output data. A linear system is capable of determining the input and output relationships through an impulse response function; however, for a nonlinear system, a higher order transfer function has to be used for this purpose. A nonlinear system can be typically modeled by Volterra or an equivalent Wiener series expansion, in which the Volterra or Wiener kernels to be estimated can fully define the system characteristics [4–6].

The kernel estimation for such nonlinear system usually requires the input signal to be a long Gaussian white noise to completely activate the underlying system. Under such conditions, Lee and Schetzen proposed a convenient cross-correlation method widely used to estimate the kernel functions [7, 8].

In several circumstances, particularly for a variety of living biosystems, input signals are constrained as a series of impulse trains instead of continuous signals, such as the Gaussian white noise [9, 10]. For instance, the auditory system is usually studied by stimulating the ear with a series of click sounds to activate the corresponding neurons in the cochlea and neural pathway to evaluate the hearing integrity [11, 12]. A well-studied impulse train for the input is a pseudorandom binary sequence called maximum length sequence (short for m-sequence), which has an important role in nonlinear system identification. The correlation property of an m-sequence is analogous to a Gaussian white noise such that to model the system by borrowing the idea of the cross-correlation method for Gaussian white noise input is possible. Hence, the binary kernels are defined using cross-correlation method for m-sequence inputs [3, 13–18].

Using the m-sequence approach, Sutter studied the binary kernel for identifying multifocal retinosystem through electroretinography and explained the visual function using the binary kernels [15]. Shi and Hecox transferred the m-sequence into an m-impulse sequence in a study on the nonlinear properties of the auditory system by measuring the electrical response from the scalp [16]. In a study on the dynamic characteristics of the primate retinal ganglion cell, Benardete and Victor developed a hybrid m-sequence allowing the summation of multiple m-sequences as input to estimate the main diagnostic kernel slice [17].

However, the binary kernel slices—derived by making use of the shift-and-product property of the m-sequence—are all laid in the first-order cross-correlation function between the m-sequence input and the system response, that is, the observed output. The specific location of any kernel slice in the cross-correlation function is determined through a complex shift function that cannot be explicitly determined. If the kernel slices are improperly arranged such that overlaps among neighboring slices occur, then the kernel estimation is inevitably distorted. A straightforward approach to solve this problem is to multiply the length of the input m-sequence, which is unfavorable for living systems with more or less time-varying property. Another approach to alleviate the overlap issue is to sparsify the impulse train of the m-sequence at risk of suffering the underestimation caused by the reduced number of available kernel slices [18].

In this study, we addressed the overlap problem through a new strategy using an inverse-repeat (IR) m-sequence. We will drive the estimation equations for the binary kernel slices corresponding to the IR m-sequence, through which the odd- and even-order kernel slices can be separately estimated and thus reduce the chance of slice overlapping. Last, a third-order nonlinear system is simulated to demonstrate the process of the proposed method.

## 2. Binary Kernel Identification for m-Sequence

### 2.1. The Properties of an m-Sequence

An m-sequence *b*[*n*] consisting of digit −1 and +1 that pseudorandomly occurred can be generated through the output of a linear circular shift register. The structure of which is determined through a primitive polynomial of degree *r*, which is also the degree of the periodical m-sequence [19, 20]. And the period or length of the m-sequence is *L* = 2^*r*^ − 1. An m-sequence is called a balanced sequence because the number of −1 is 2^*r*−1^, which is just one more than the number of +1, that is, 2^*r*−1^ − 1. Two crucial properties of the m-sequence used in the present study are as follows.

(i) Shift-and-product property

(1) bnbn−j1⋯bn−j1−⋯−jp−1=bn−fp,iiiiiiiiiiiiiiiiiiiiiiiiiiiiiiiiiiiiiiiiiiiiiiiiiiiiiiij1,…,jp−1≠0,

where *f*_*p*_ = *f*(*j*_1_,…, *j*_*p*−1_) is referred to as* shift function* representing the circular shift lag of the m-sequence *b*[*n*]. The exact value of a shift function depends on the shifting lags of the m-sequences to be multiplied. This property indicates that the product of the m-sequences with different circular shifts is also the same m-sequence circular shifting to a lag determined by a shift function *f*_*p*_ that is unknown* a priori*. A straightforward approach to calculate the specific value of a shift function is to compare bit-by-bit the original m-sequence *b*[*n*] and the resulting *k*-bit shifting version *b*[*n* − *k*] until *b*[*n*] = *b*[*n* − *k*], such that *f*_*p*_ = *k*.

For *j*_1_ = ⋯ = *j*_*p*−1_ = 0, that is, the product of the same *j*  m-sequences, it yields

(2) $$\begin{array}{c} b^{j}\left[n\right]=\left\{\begin{array}{cc} b\left[n\right], & j=\text{odd}\\ \left\{1\right\}, & j=\text{even}, \end{array}\right. \end{array}$$

where {1} denotes an all-one sequence—all members of the sequence are 1s.

Equation (2) provides an exception for the shift-and-product property that an all-one sequence instead of an m-sequence is produced under a certain condition when multiplying the same m-sequences. When more than two different m-sequences are multiplied, we have another exception for the shift-and-product property:

(3) bnbn−j1⋯bn−j1−⋯−jp−1=1,iiiiiiiiiiiiiiiiiiiiif  fp−1=j1+⋯+jp−1, p≥3.

Equation (3) implies that when dealing with higher-order kernels (*p* ≥ 3), the m-sequence should be selected with caution in case invalid results occur for the kernel estimation.

(ii) The autocorrelation function *ϕ*_*bb*_[*n*] is a periodical real value function with the same minimal period of *L*; that is,

(4) ϕbbn=1L∑τ=0L−1b[τ]b[τ+n]=1n=0−1Lotherwise, hhhihhhhh0≤n<L.

In analogy to the Gaussian white noise method, this autocorrelation property of (4) is important to account for the derivation of the m-sequence in identifying a nonlinear system.

### 2.2. From Volterra Kernels to Binary Kernels

The output *y*[*n*] of a general *Q*th-order nonlinear dynamic system in response to the input *b*[*n*] can be modeled by a Volterra series expansion [5, 21],

(5) $$\begin{array}{c} y\left[n\right]=\overset{Q}{\underset{p=0}{\sum }}H_{p}b\left[n\right], \end{array}$$

where ℋ_*p*_ is called the *p*th-order Volterra operator which is defined as

(6) Hpbn =∑k1=0M−1⋯∑kp=0M−1hpk1,…,kpbn−k1⋯bn−kp,

where *M* represents the* memory length* of the dynamic system, and *h*_*p*_ represents the *p*th-order* Volterra kernel*.

To estimate the Volterra kernel for Gaussian white noise, input is not theoretically feasible for the difficulty of nonorthogonality. Instead, it is preferred to estimate the* Wiener kernel* after the Gram-Schmidt orthogonal process on the Volterra series expansion [21]. This method can be extended to deal with m-sequence input yielding the so-called* binary kernel* estimation [13, 16]. A *p*th-order binary kernel is given by

(7) $$\begin{array}{c} w_{p}\left[k_{1},k_{2},\ldots ,k_{p}\right]=\frac{1}{p!}\phi_{b^{p}y}\left[k_{1},k_{2}\ldots ,k_{p}\right], \end{array}$$

where the *p*th-order cross-correlation of input *b*[*n*] and output *y*[*n*] is

(8) ϕbpy[k1,k2…,kp] =1L∑i=0L−1y[i]b[i−k1]b[i−k2]⋯b[i−kp].

Let *k* = *k*_1_ and  *l*_*i*_ = *k*_*i*+1_ − *k*_*i*_, and then (7) and (8) become

(9)   wp[k,k+l1,…,k+l1+⋯+lp−1] =1p!ϕbpy[k,k+l1…,k+l1+⋯+lp−1],

(10) ϕbpy[k,k+l1…,k+l1+⋯+lp−1] =1L∑i=0L−1y[i]b[i−k]  ×b[i−k−l1]⋯b[i−k−l1−⋯−lp−1].

According to the shift-and-product property, (10) becomes

(11) ϕbpy[k,k+l1,…,k+l1+⋯+lp−1] =1L∑i=0L−1y[i]b[i−k−f(l1,…,lp−1)] =ϕbyk+fp,

which transfers the multivariable correlation function *ϕ*_*b*^*p*^*y*_[*k*, *k* + *l*_1_,…, *k* + *l*_1_ + ⋯+*l*_*p*−1_] into a single variable cross-correlation function *ϕ*_*by*_[*k* + *f*_*p*_]. Substituting (11) to (9) yields

(12) $$\begin{array}{c} w_{p}\left[k,k+l_{1},\ldots ,k+l_{1}+\cdots +l_{p-1}\right]=\frac{1}{p!}\phi_{by}\left[k+f_{p}\right]. \end{array}$$

**Figure 1:**
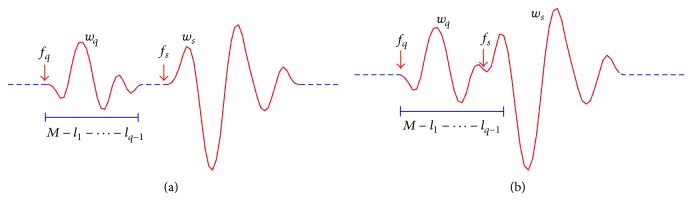
Illustration of overlapping condition for two arbitrary kernel slices. A piece of cross-correlation function contains *q*th- and *s*th-order kernel slices, that is, *w*_*s*_ and *w*_*q*_, respectively. Corresponding shift functions *f*_*q*_ and *f*_*s*_ designate the beginnings of kernel slices. The duration of *w*_*q*_ is dependent on memory length *M* and sum of shift lags (*l*_1_ + ⋯+*l*_*q*−1_). According to (14), nonoverlap and overlap cases are presented in (a) and (b), respectively.

Given *l*_1_, *l*_1_,…, *l*_*p*−1_, (12) presents a portion of the kernel function values along the diagonal and subdiagonal dimensions and is called binary* kernel slice*. Considering the confinement for the independent variables for *w*_*p*_, the kernel slice is probably unable to completely cover the true binary kernel along this dimension. Given the memory length similar to (6), all variables for *p*th-order kernel slice must be in the range of the memory length *M*; hence,

(13) $$\begin{array}{c} l_{p-1}\in \left[1,M\right);\\ l_{p-2}\in \left[1,M-l_{p-1}\right);\ldots ;l_{1}\in \left[1,M-l_{2}-\cdots -l_{p-1}\right);\\ k\in \left[0,M-l_{1}-\cdots -l_{p-1}\right), \end{array}$$

suggesting that *w*_*p*_ is defined through the cross-correlation function *ϕ*_*by*_ between *f*_*p*_ and *f*_*p*_ + (*M* − *l*_1_ − ⋯−*l*_*p*−1_). Therefore, if the shift functions of two neighboring slices satisfy

(14) $$\begin{array}{c} f_{s}-f_{q}\leq M-l_{1}-\cdots -l_{q-1}, \end{array}$$

that is, the interval between an arbitrary kernel slice of order *s* and another kernel slice of order *q* is less than the length of the prior slice, then a slice overlap occurs. The kernel slices overlapping condition is illustrated in Figure 1.

## 3. Estimate Binary Kernel Slices Using IR m-Sequence

### 3.1. The Properties of IR m-Sequence

The IR m-sequence of an m-sequence *b*[*n*] is defined as

(15) $$\begin{array}{c} a\left[n\right]=b\left[n\right]u\left[n\right],\;0\leq n<2L, \end{array}$$

where *u*[*n*] = {(−1)^*n*^} is a square wave-like function with alternates 1 and −1. Several useful properties of an IR m-sequence are given as follows [22, 23].

(i) The minimal period of *a*[*n*] is twice as that of *b*[*n*], and *a*[*n*] satisfies the inverse repeatability, that is,

(16) $$\begin{array}{c} a\left[n\right]=-a\left[n+L\right],\;0\leq n<L. \end{array}$$

(ii) The output *y*[*n*] of a nonlinear system in response to the input *a*[*n*] can also be modeled by Volterra series expansion. If we dichotomize *y*[*n*] into an odd-order response *y*_*o*_[*n*] = ∑ℋ_*p*_ *b*[*n*],  *p* = odd, and even-order response *y*_*e*_[*n*] = ∑ℋ_*p*_ *b*[*n*], *p* is even, they satisfy,

(17) yon=−yon+L  yen=yen+L,iiiiiiiiiiiiiiiiiiiiiiiiiiiiiiiiiiiiiiiiiiiiiiiii0≤n<L.

(iii) The cross-correlation function of *a*[*n*] and *b*[*n*] is zero, that is,

(18) $$\begin{array}{c} \phi_{ab}\left[n\right]=\frac{1}{2L}\overset{2L-1}{\underset{\tau =0}{\sum }}a\left[\tau \right]b\left[\tau +n\right]=0,\;0\leq n<2L. \end{array}$$

(iv) The autocorrelation of *a*[*n*] is

(19) $$\begin{array}{c} \phi_{aa}\left[n\right]=\left\{\begin{array}{cc} -\phi_{bb}\left[n\right], & n=\text{odd}\\ \phi_{bb}\left[n\right], & n=\text{even}. \end{array}\right. \end{array}$$

If the minimal period of the m-sequence *L* is odd, then *ϕ*_*aa*_[*n*] = −*ϕ*_*aa*_[*n* + *L*]; that is, *ϕ*_*aa*_[*n*] is also inversely repeated. The proof of property (iv) is given in Appendix A.

(v) IR m-sequence *a*[*n*] also has the following shift-and-product property,

(20) a[n]a[n−j1]⋯a[n−j1−⋯−jp−1] =−1j1+⋯+(j1⋯+jp−1)+fpa[n−fp],p=odd−1j1+⋯+(j1⋯+jp−1)b[n−fp],p=even.

The proof of property (v) can be found in Appendix B. In reference to Volterra series expansion for a nonlinear system, this property for an IR m-sequence implies that the higher-order Volterra operators can be separated into odd- and even-order cases.

### 3.2. Odd- and Even-Order Kernel Slices for IR m-Sequences

Based on the above properties of the IR m-sequences, we can derive the binary kernel slices in response to an IR m-sequence. Let *a*[*n*] be the input of the system, and then the output of the *Q*th-order nonlinear system expressed in terms of Volterra series expansion is

(21) $$\begin{array}{c} y\left[n\right]=\overset{Q}{\underset{p=0}{\sum }}H_{p}a\left[n\right]. \end{array}$$

Again, let *k* = *k*_1_ and *l*_*i*_ = *k*_*i*+1_ − *k*_*i*_, and, using IR m-sequence property (v), the Volterra operator on the input *a*[*n*] becomes

(22) Hpan=∑k=0M−1⋯∑lp−1=−M+1M−1hp[k,…,k+l1+⋯+lp−1]  ×an−k⋯an−k−l1−⋯−lp−1.

Exploiting (20) for the property (v) of the IR m-sequences, we can derive that the cross-correlation function between the m-sequences *b*[*n*] and odd-order Volterra series term ℋ_*p*_ *a*[*n*], (*p* = odd), is zero and that the cross-correlation function between IR m-sequences *a*[*n*] and even-order Volterra series term ℋ_*p*_ *a*[*n*] (*p* = even) is also zero; that is,

(23) $$\begin{array}{c} \phi_{bH_{p}}\left[n\right]=\frac{1}{2L}\overset{2L-1}{\underset{\tau =0}{\sum }}b\left[\tau \right]H_{p}a\left[\tau +n\right]=0,\;p=\text{odd}\\ \phi_{aH_{p}}\left[n\right]=\frac{1}{2L}\overset{2L-1}{\underset{\tau =0}{\sum }}a\left[\tau \right]H_{p}a\left[\tau +n\right]=0,\;p=\text{even}. \end{array}$$

The proof for (23) is given in Appendix C.

We can derive the *p*th-order cross-correlation for IR m-sequence input *a*[*n*] with the same approach as that of getting (7)–(12) to give the expression of the *p*th-order kernel slices as

(24) wp[k,k+l1,…,k+l1+⋯+lp−1] =1p!ϕapy[k,k+l1,…,k+l1+⋯+lp−1],

where, according to the definition of cross-correlation function, the right side member is

(25) ϕapy=12L∑i=02L−1y[i]a[i−k] ×a[i−k−l1]⋯a[i−k−l1−⋯−lp−1].

By exploiting the property (v) of an IR m-sequence {*a*[*n*]}, (25) can be separately formulated for *p* being odd and even,

(26) $$\begin{array}{c} \phi_{a^{p}y}=\left\{\begin{array}{cc} {\left(-1\right)}^{l_{1}+\cdots +(l_{1}\cdots +l_{p-1})+f_{p}}\phi_{ay}\left[k+f_{p}\right], & p=\text{odd}\\ {\left(-1\right)}^{l_{1}+\cdots +(l_{1}\cdots +l_{p-1})}\phi_{by}\left[k+f_{p}\right], & p=\text{even}. \end{array}\right. \end{array}$$

Therefore, (24) becomes

(27) $$\begin{array}{c} w_{p}=\left\{\begin{array}{cc} {\left(-1\right)}^{l_{1}+\cdots +(l_{1}\cdots +l_{p-1})+f_{p}}\frac{1}{p!}\phi_{ay}\left[k+f_{p}\right], & p=\text{odd}\\ {\left(-1\right)}^{l_{1}+\cdots +(l_{1}\cdots +l_{p-1})}\frac{1}{p!}\phi_{by}\left[k+f_{p}\right], & p=\text{even}. \end{array}\right. \end{array}$$

Equation (27) indicates that the kernel slices of odd-order exist exclusively in the cross-correlation function between the IR m-sequence *a*[*n*] and output *y*[*n*], and likewise, kernel slices of even-order exist exclusively in the cross-correlation function between the m-sequence *b*[*n*] and output *y*[*n*].

According to (21), which expresses the output *y*[*n*] in terms of Volterra series expansion, we can rewrite the left-side member of (27) as *ϕ*_*ay*_[*n*] = *ϕ*_*a*ℋ_0__[*n*] + *ϕ*_*a*ℋ_1__[*n*]+⋯+*ϕ*_*a*ℋ_*Q*__[*n*]. Moreover, (23) does not state even-order *ϕ*_*a*ℋ_*i*__ in the odd-order *ϕ*_*ay*_[*n*], or the even-order Volterra kernels are also excluded from the odd-order binary kernel slices. The similar argument can be obtained for the case of even-order binary kernel slices.

## 4. Simulation Results and Discussion

### 4.1. Nonlinear Wiener Model Generation

**Figure 2:**
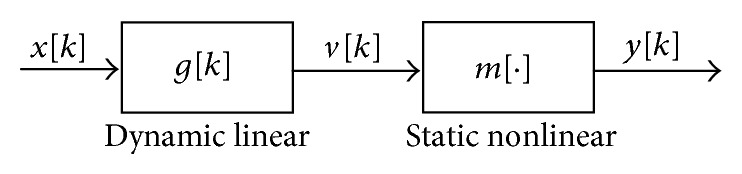
Weiner model representing a general nonlinear dynamic system in cascade form.

A general nonlinear dynamic system can be represented by a Wiener model consisting of two subsystems in cascade form [24]. This system consists of a dynamic linear subsystem *g*[*n*] followed by a static nonlinear subsystem *m*[·], as shown in Figure 2. The output of the dynamic subsystem *g*[·] is

(28) $$\begin{array}{c} v\left[n\right]=\overset{M-1}{\underset{k=0}{\sum }}x\left[k\right]g\left[n-k\right], \end{array}$$

where *M* represents the memory length of the system.

For simplicity, we simulate a three-order nonlinear system expressed in the second module *m*[·]. Then, the output is

(29) $$\begin{array}{c} y\left[v\right]=a_{0}+a_{1}v+a_{2}v^{2}+a_{3}v^{3}. \end{array}$$

Therefore, the output of the whole nonlinear system is expressed as a high-order convolution of the input and the Volterra kernels as

(30) yn=a0+a1∑k=0M−1g[k]x[n−k]+a2∑k1=0M−1 ∑k2=0M−1g[k1]g[k2]x[n−k1]x[n−k2]+a3∑k1=0M−1 ∑k2=0M−1 ∑k3M−1gk1gk2gk3xn−k1×xn−k2xn−k3.

From (3), the following relationships between Volterra kernels and the parameter model hold

(31) $$\begin{array}{c} h_{0}=a_{0},\\ h_{1}\left[k\right]=a_{1}g\left[k\right],\\ h_{2}\left[k_{1},k_{2}\right]=a_{2}g\left[k_{1}\right]g\left[k_{2}\right],\\ h_{3}\left[k_{1},k_{2},k_{3}\right]=a_{3}g\left[k_{1}\right]g\left[k_{2}\right]g\left[k_{3}\right]. \end{array}$$

Let *k* = *k*_1_, *l*_1_ = *k*_2_ − *k*_1_, and *l*_2_ = *k*_3_ − *k*_2_, and then the binary kernel slices can be associated with the Volterra kernel for this model [12] which is

(32) w0=a0−1L∑k=0M−1h1[k]+∑k=0M−1 ∑l=−M+1M−1h2[k,k+l]fffffhffff+∑k=0M−1 ∑l1=−M+1M−1 ∑l2=−M+1M−1h3k,k+l1,k+l1+l2,w1[k]=h1[k]+h3[k,k,k]+3∑l=−M+1l≠0M−1h3k,k+l,k+l,w2k,k+l=h2k,k+l,w3k,k+l1,k+l1+l2=h3k,k+l1,k+l1+l2.

In the present study, the memory length *M* = 10, impulse response function *g*[*n*] = *e* ^−*n*/2^ · sin(2*πn*/5), and the third-order polynomial coefficients *a*_0_ = 0, *a*_1_ = 1.4, *a*_2_ = 5, and *a*_3_ = 10 are set.

We assume that *S* represents the total length of all the kernel slices spread along the cross-correlation function, as illustrated in Figure 1. To avoid the overlap among these slices, at least the length of the cross-correlation function, *L*  ( = 2^*r*^ − 1), should be larger than *S*, indicating that the degree of the m-sequence should satisfy the condition,

(33) $$\begin{array}{c} r\geq \left\lfloor \text{lo}{\text{g}}_{2}\left(S+1\right)\right\rfloor , \end{array}$$

where ⌊(·)⌋ denotes the integer part of (·).

According to (13), the shift lags are set to start with minimal values min⁡(*l*_*i*_) = 1, and we can obtain the total slice length for this three-order nonlinear system as *S* = 175 for *M* = 10, and the degree of the m-sequence should be *r* ≥ 8 to avoid the slice overlap.

### 4.2. Comparison of m- and IR m-Sequence Inputs

**Figure 3:**
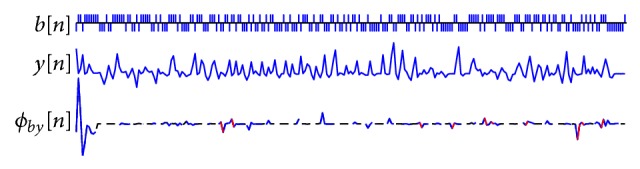
Estimation process of kernel slices in cross-correlation function between m-sequence input and output from nonlinear system in the present study.

We select an arbitrary eight-degree m-sequence in this identification instance.  Figure 3 shows the binary input m-sequence *b*[*n*], output signal *y*[*n*], and cross-correlation *ϕ*_*by*_[*n*]. The estimated kernel slices are plotted in solid trace superimposed on the cross-correlation signal (in dashed trace). The red trace segments indicate the occurrence of slice overlaps. According to the overlap condition defined in (14), a total of 10 overlaps occurred in the cross-correlation function.

**Figure 4:**
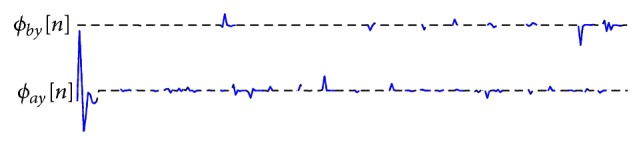
Odd- and even-order kernel slices separately displayed in two cross-correlation functions for IR m-sequence method.

The kernel slices estimated for the IR m-sequence input *a*[*n*] are shown in Figure 4. The odd- and even-order kernel slices are separated into two traces of the cross-correlation, as indicated in (27). All slices are notably separated without any overlap.

**Figure 5:**
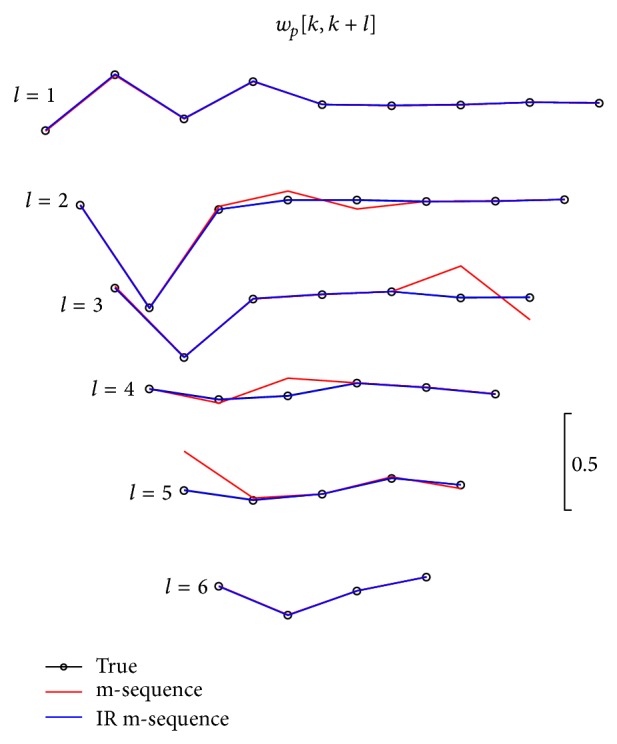
Second-order kernel slices extracted from cross-correlation function. True values are also plotted in black traces with circle markers, and estimated kernel slices are plotted in red and blue traces for m- and IR m-sequence methods, respectively.

**Figure 6:**
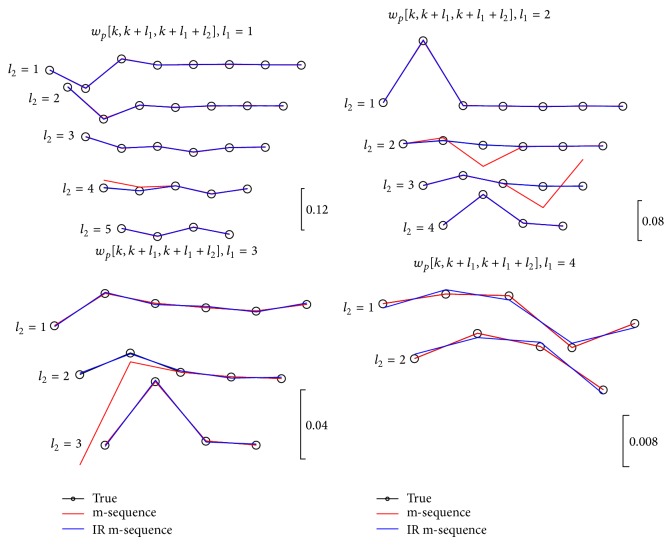
Third-order kernel slices extracted from cross-correlation functions. True kernel slices are plotted in black traces with circle markers, and estimated kernel slice values are plotted in red and blue traces for m- and IR m-sequence methods.

The distortion severity due to overlapping does not display in Figure 3 without the true kernels. Therefore, we extract and compare the estimated and the true kernel slices for the second- and third-order systems in Figures 5 and 6, respectively. These are two-dimensional illustration, where the kernel slices with different lags are plotted by projecting them to the main-diagonal direction of that order. Notably, the length of the kernel slices decreases with increasing values of shift lags. The kernel slices with the length less than four data samples were excluded in Figures 5 and 6 for less practical significance.

Six second-order kernel slices estimated by the m-sequence (in read traces) and proposed IR m-sequence (in blue traces) methods are shown in Figure 5. The true or theoretical kernel slices are also given as a benchmark. Four kernel slices determined using the m-sequence evidently deviated from the true values; however, no such distortion was observed for IR m-sequence method.

A series of third-order kernel slices are shown in Figure 6. Similar to Figure 5, IR m-sequence method shows more consistent results with the true values. However, the third-order kernel slices for *l*_1_ = 4 show a slightly opposite behavior that needs to be accounted for, because no overlap occurs at these affected kernel slices. This inconsistency is only observable at a significantly small scale (see the amplitude scales in Figure 6) caused by approximating the orthogonality of the IR m-sequence and has no appreciable effects upon practical application. Alternatively, increasing the length of the m-sequence will alleviate this problem. Further analysis of this intrinsic property is beyond the scope of the present study and more details can be found in [25, 26].

## 5. Conclusion

The shift-and-product property of the m- and IR m-sequence is crucial in the derivation of new properties to address the overlap problem for short-length m-sequence. It is apparently not beneficial to use a redundantly higher order m-sequence or to sacrifice the number of the estimated slices using sparse m-sequence. In this study, we alternatively propose an approach by introducing the IR m-sequence. We provide and prove several relevant properties of the IR m-sequence allowing the estimation of the binary kernel slices. By examining the special shift-and-product properties of the IR m-sequence derived for odd and even shifts, the odd- and even-order kernel slices can be separately represented in the cross-correlation functions, such that the chance of overlapping significantly decreases. Furthermore, this separation will be useful in some special applications where only odd- or even-order kernel slices might be of significant interest.

## Appendices

### A. The Proof of (19)

Given an IR m-sequence *a*[*n*], using (15) which relates it to the corresponding m-sequence *b*[*n*], we have the autocorrelation function

(A.1) ϕaan=12L∑k=02L−1a[k]a[k+n]  =12L∑k=02L−1bkbk+nukuk+n.

Since *b*[*n*] and *u*[*n*] are uncorrelated, (A.1) becomes

(A.2) ϕaan=12L∑k=02L−1bkbk+n×12L∑k=02L−1ukuk+n=ϕbb[n]ϕuu[n].

Since *u*[*n*] = {(−1)^*n*^}, it is easy to get that *ϕ*_*uu*_[*n*] = *u*[*n*] = {(−1)^*n*^}, and then (19) is proved.

### B. The Proof of (20)

According to the relationship between *a*[*n*] and *b*[*n*] in (15), and the shift-and-product property in (1), the shift-and-product of *a*[*n*] becomes

(B.1) anan−j1⋯an−j1−⋯−jp−1 =bn−fpunun−j1⋯un−j1−⋯−jp−1.

Since *u*[*n*] = {(−1)^*n*^},  *n* = 0,1,…, 2*L* − 1, we get

(B.2) u[n]u[n−j1]⋯u[n−j1−⋯−jp−1] =−1j1+⋯+(j1⋯+jp−1)u[n],p=odd−1j1+⋯+(j1⋯+jp−1){1},p=even.

Equation (B.1) is rewritten as

(B.3) a[n]a[n−j1]⋯a[n−j1−⋯−jp−1] =−1j1+⋯+(j1⋯+jp−1)u[n]b[n−fp],p=odd−1j1+⋯+(j1⋯+jp−1)b[n−fp],p=even.

Because the relationship between *u*[*n*] and its circular shift to *k* lag is *u*[*n*] = (−1)^*k*^ *u*[*n* − *k*], then the left-side member of (B.3) for odd number *p* becomes

(B.4) unbn−fp=−1fpu[n−fp]b[n−fp]=−1fpan−fp.

Substitute it into (B.3), and then (20) is proved.

### C. The Proof of (23)

According to the shift-and-product property of IR m-sequence in (20), the *p*th-order Volterra series term becomes

(C.1) Hpa[n]=∑k=0M−1⋯∑lp−1=−M+1M−1hp[k,…,k+l1+⋯+lp−1] ×−1l1+⋯+(l1⋯+lp−1)+fp ×an−k−fp,p=odd∑k=0M−1⋯∑lp−1=−M+1M−1hp[k,…,k+l1+⋯+lp−1] ×−1l1+⋯+l1⋯+lp−1 ×bn−k−fp,p=even.

If *p* is odd, the cross-correlation between ℋ_*p*_ *a*[*n*] and *b*[*n*] is

(C.2) ϕbHpn=12L∑τ=02L−1b[τ]Hpa[τ+n]=  ∑k=0M−1⋯∑lp−1=−M+1M−1hpk,…,k+l1+⋯+lp−1 ×−1l1+⋯+(l1⋯+lp−1)+fp ×12L∑τ=02L−1aτ+n−k−fpbτ  =∑k=0M−1⋯∑lp−1=−M+1M−1hp[k,…,k+l1+⋯+lp−1] ×−1l1+⋯+l1⋯+lp−1+fp ×ϕabn−k−fp.

If *p* is even, by the similar process, the cross-correlation between ℋ_*p*_ *a*[*n*] and *a*[*n*] is

(C.3) ϕaHpn=∑k=0M−1⋯∑lp−1=−M+1M−1hp[k,…,k+l1+⋯+lp−1]×−1l1+⋯+l1⋯+lp−1×ϕabn−k−fp.

According to the property (iii) of IR m-sequence in (18) which tells that the cross-correlation between *a*[*n*] and *b*[*n*] is zero, we can get that both (C.2) and (C.3) are zero. Equation (23) is thus proved.
